# Risk Factors for Central and Branch Retinal Vein Occlusion: A Meta-Analysis of Published Clinical Data

**DOI:** 10.1155/2014/724780

**Published:** 2014-06-09

**Authors:** Petr Kolar

**Affiliations:** University Eye Clinic, Masaryk University Brno and University Hospital Brno, Jihlavska 20, 625 00 Brno, Czech Republic

## Abstract

Retinal vein occlusion (RVO) is a major cause of vision loss. Of the two main types of RVO, branch retinal vein occlusion (BRVO) is 4 to 6 times more prevalent than central retinal vein occlusion (CRVO). A basic risk factor for RVO is advancing age. Further risk factors include systemic conditions like hypertension, arteriosclerosis, diabetes mellitus, hyperlipidemia, vascular cerebral stroke, blood hyperviscosity, and thrombophilia. A strong risk factor for RVO is the metabolic syndrome (hypertension, diabetes mellitus, and hyperlipidemia). Individuals with end-organ damage caused by diabetes mellitus and hypertension have greatly increased risk for RVO. Socioeconomic status seems to be a risk factor too. American blacks are more often diagnosed with RVO than non-Hispanic whites. Females are, according to some studies, at lower risk than men. The role of thrombophilic risk factors in RVO is still controversial. Congenital thrombophilic diseases like factor V Leiden mutation, hyperhomocysteinemia and anticardiolipin antibodies increase the risk of RVO. Cigarette smoking also increases the risk of RVO as do systemic inflammatory conditions like vasculitis and Behcet disease. Ophthalmic risk factors for RVO are ocular hypertension and glaucoma, higher ocular perfusion pressure, and changes in the retinal arteries.

## 1. Introduction

Retinal vein occlusion (RVO) is the second most common retinal vascular disease after diabetic retinopathy. There are two types of RVO: branch retinal vein occlusion (BRVO) and central retinal vein occlusion (CRVO). BRVO is more common than CRVO. In BRVO there is occlusion of a branch of the retinal vein system, while, in CRVO, occlusion is located in the central retinal vein [1, 2]. Hayreh divided RVO into three types: (1) BRVO is divided further into major BRVO and macular BRVO; (2) CRVO is divided into ischemic and nonischemic types; and (3) hemi-CRVO with involvement of only one half of the retina surface and like CRVO is divided into ischemic and nonischemic types [3].

RVO is more prevalent in men than women and is more frequent in older age (over 65 years). The International Eye Disease Consortium published a paper in 2010 that reported the prevalence of RVO in the USA, Europe, Asia, and Australia [4]. The combined pooled data contained 68,751 individuals from 15 studies, with participants' ages ranging from 30 to 101 years. The prevalence of RVO was 5.20 per 1000 (confidence interval (CI), 4.40–5.99) for any RVO, 4.42 per 1000 (CI, 3.65–5.19) for BRVO, and 0.80 per 1000 (CI, 0.61–0.99) for CRVO [4]. This shows that BRVO is 4 times more common than CRVO. In one study, the prevalence was found to vary by race/ethnicity and increased with age but did not differ according to gender [4]. This provides summary data on the prevalence of RVO and suggests that approximately 16 million people may have this condition. Research on preventive and treatment strategies for this sight threatening eye disease is needed [4]. The Beijing Eye Study states, on the basis of a sample of 4439 patients, that the 10-year incidence of BRVO is 1.6 per 100 subjects (43 subjects (88% of patients with RVO); 44 eyes), and the incidence of CRVO was 0.3 per 100 persons. The mean age (in 2001) was 56.9 ± 10.2 years (range, 40–80 years), and mean refractive error was −0.64 ± 2.30 diopters (range, −10.25 to +2.75 diopters). Macular edema was present in 30% of BRVO cases [5].

Its pathogenesis is still not completely understood. The condition may be due to a combination of three systemic changes known as Virchow**'**s triad: (1) hemodynamic changes (venous stasis), (2) degenerative changes of the vessel wall, and (3) blood hypercoagulability [6].

Due to its multifactorial nature, treatment of RVO is still a challenge. To date, however, no causal treatment has been shown in large randomized studies to be effective [6]. A number of therapies have been assessed in the treatment of RVO (laser photocoagulation, intravitreal steroids and anti-VEGF agents, surgical procedures—pars plana vitrectomy and systemic treatments—hemodilution, anticoagulation therapy, and fibrinolysis) [6].

## 2. Risk Factors for RVO

RVO has many known ophthalmic and systemic risk factors. As we know from its pathogenesis, Virchow's triad is important: hemodynamic changes (venous stasis), degenerative changes of the vessel wall, and blood hypercoagulability. Known risk factors are summarized in Table 1.

The most recognized risk factors for RVO are age and systemic vascular disorders. In over half of the cases, the age of onset is over 65 years. However, patients under 45 can also develop an RVO [6].

### 2.1. Systemic Risk Factors for RVO

Systemic diseases such as HTN, HLD, and DM are very strongly associated with the development of RVO [8]. The data of published studies suggest that 48% of RVO is connected to NTH, 20% to HLD, and 5% to DM [9]. Cigarette smoking has also been linked to RVO [10]. The Diabetes Control and Complications Study (DCCT) and Blue Mountains Study also reported that HTN, HLD, arteriosclerosis, and DM are risk factors for RVO [11, 12]. Schmidt on small sample of patients with RVO in combination with retinal artery occlusion (RAO) described a variety of systemic risk factors. Systemic risk factors were present in 11 out of 14 subjects (mainly HTN 8x, HLD 3x, and chronic smoking 3x) [13].

## 3. Central Retinal Vein Occlusion (CRVO)

### 3.1. Systemic Risk Factors for CRVO

Data have also recently been published on risk factors connected with CRVO [14]. The authors identified risk factors associated with CRVO among a diverse group of patients throughout the United States. A total of 494 165 subjects met the study inclusion criteria. The mean age of this CRVO study population was 69.6 years. CRVO were diagnosed in 1302 subjects; 667 (51.2%) were female, and the racial distribution included 1017 whites (78.1%), 94 blacks (7.2%), 51 Latinos (3.9%), 13 Asians (1.0%), 7 of other races (0.54%), and 120 whose race was not documented (9.2%) [14]. There were no gender differences. The white race predominated.

### 3.2. Socioeconomic Factors for CRVO

American blacks had increased risk by 58% of CRVO being diagnosed compared with non-Hispanic whites (adjusted hazard ratio (HR), 1.58; 95% confidence interval (CI), 1.25–1.99). There was no statistically significant increased risk for CRVO in Latinos and Asians compared to non-Hispanic whites. Women were 25% less likely than men to have CRVO (HR, 0.75; 95% CI, 0.66–0.85) [14].

### 3.3. Systemic Diseases Associated with CRVO

The majority of people diagnosed with CRVO had more than 1 component of the metabolic syndrome, defined as the presence of coexisting HTN, DM, and HLD [14]. Metabolic syndrome was diagnosed in 487 subjects with CRVO (37.4%). By comparison, 142 101 individuals (28.8%) who were not diagnosed with CRVO had the metabolic syndrome [14]. Further systemic risk factors for CRVO identified in this study instead of the metabolic syndrome were separately diagnosed HTN, DM, HLD, further, peripheral artery disease (PAD), myocardial infarction (MI), deep venous thrombosis (DVT), pulmonary embolism (PE), and a hypercoagulable stage. Statistically significant differences were found in all the above-mentioned conditions between two groups (subjects with and without CRVO). Subjects with diagnosed CRVO were more likely to have all the above systemic risk factors. The authors used a regression model to assess the relationship between components of the metabolic syndrome (HTN, DM, and HLD) individually and in combination with the risk of CRVO. HTN increases the risk of CRVO by 66%. Subjects with DM alone (no HTN or HLD) or HLD alone (no DM or HTN) had no increased risk of CRVO. Individuals with all 3 metabolic syndrome components had a 58% increased risk of developing CRVO relative to those with none of these conditions [14].

Risk factors connected with vascular diseases include patients with prior stroke (44% increased risk) (HR, 1.44; 95% CI, 1.23–1.68). Those with PAD had a 15% increased hazard of CRVO (HR, 1.15; 95% CI, 1.00–1.33). Risk of CRVO was very strongly increased in subjects with proven hypercoagulable stage. These subjects had 145% higher risk of CRVO (HR, 2.45; 95% CI, 1.40–4.28). Surprisingly, subjects with DVT and PE had no increased risk of CRVO (HR, 0.86; 95% CI, 0.55–1.34). A prior MI actually had decreased hazard of being diagnosed with CRVO (HR, 0.72; 95% CI, 0.57–0.92). Use of anticoagulants surprisingly did not decrease the risk of development of CRVO (*P* = 0.60) [14].

### 3.4. Severity of Systemic Disease

One study examined whether severity of HTN and DM had any impact on the risk of developing CRVO. The authors found that subjects with noncomplicated HTN had a 36% increased risk of developing CRVO. Moreover, subjects with advanced HTN had 92% increased risk of CRVO [14]. Participants with no end-organ damage caused by DM were not at increased risk for CRVO (HR, 0.87; 95% CI, 0.73–1.04) but those with end-organ damage from DM had a 53% increased hazard of CRVO (HR, 1.53; 95% CI, 1.28–1.84) DM [14].

Can we generalize the results of this survey regarding the risk of developing CRVO? The authors emphasized that HTN is a major risk factor. Other risk factors are less clearly defined but we can say that advanced DM also increases the risk of CRVO [14]. For both conditions (HTN and DM), common is the increased risk of systemic arteriosclerosis, which is generally considered the main pathophysiological component of developing RVO. Advanced DM is risk factor for CRVO, compared to DM without complications that do not increase the risk of CRVO. HLD is not a risk factor for developing CRVO. The presence of other arterial diseases such as PAD and vascular cerebral stroke increases the risk of CRVO. The role of hypercoagulability is still controversial. Coagulopathies like factor V deficiency are more prevalent in younger subjects. Socioeconomic factors play a role in blacks [14]. The higher risk for CRVO in blacks compared to whites can be further attributed to the higher prevalence of glaucoma in blacks [14].

### 3.5. Ocular Risk Factors for CRVO

One main ocular risk factor associated with CRVO is glaucoma [11]. The Chinese study published by Zhou in 2013 (10-year incidence of RVO from 2001 to 2011) updated the association of main risk factors for RVO in a Chinese population. Very strong association with RVO was shown with lower ocular pressure resulting in venous stasis (part of Wirchow's triad) (*P* = 0.02). In contrast, increased RVO risk was not associated with refractive error (*P* = 0.74), high myopia (above 8 diopters) (*P* = 0.55), intraocular pressure measured in 2001 (*P* = 0.39), 2006 (*P* = 0.85), and 2011 (*P* = 0.99), central cornea thickness (*P* = 0.23), anterior chamber depth (*P* = 0.07), lens thickness (*P* = 0.56) or axial length (*P* = 0.26), or glaucoma in 2006 (*P* = 0.30) [5]. Lower ocular pressure may be connected either with glaucoma or with systemic risk factors like HLD, DM, and arteriosclerosis.

## 4. Branch Retinal Vein Occlusion (BRVO)

The recent published meta-analysis of Jaulim et al. showed pooled data on main and subsidiary risk factors connected to BRVO [15]. BRVO was, as stated before, 4 to 6 times more common than CRVO. Meta-analysis showed a prevalence of BRVO in 0.5–1.2% [3, 4, 10, 12, 16, 17]. Advancing age is also a very important risk factor for RVO. The meta-analysis by Rogers et al. [4] showed a 1.57 per 1,000 prevalence of BRVOs in 40-to-49-year olds (4.58 per 1,000), in 50-to-59-year olds (11.11 per 1,000), and in 60-to-69-year olds, 12.76 per 1,000 in 70-to-79-year olds, and 10.32 per 1,000 in those older than 80 years. The prevalence in subjects older than 80 is 7 times higher than people from 40 to 49 years [4].

The main pathogenic mechanism for development of BRVO is arterial stiffness that causes venous compression in the common adventitial sheath [17–19].

### 4.1. Risk Factors for BRVO

Systemic vascular diseases like HTN, HLD, and PAD and metabolic diseases like DM are very strongly connected with BRVO. A meta-analysis showed that, in BRVO, the odds ratio for HTN is 3.0 (95% CI: 2.0–4.4), for HLD 2.3 (95% CI: 1.5–3.5), and for diabetes mellitus (DM) 1.1 (95% CI: 0.8–1.5) [20]. In a study published by Lam et al. risk factors for developing BRVO in 60 patients younger than 50 years were very similar to those in older people (HTN, HLD, and high body mass index) [21]. It is not completely clear what role thrombophilia plays in BRVO pathogenesis. Blood abnormalities play a controversial role in the pathogenesis of BRVO; erythrocyte volume, level of fibrinogen, and hematocrit appear to be important [22]. Recent meta-analyses of RVO and thrombophilic factors by Zhou et al., J. Rehak and M. Rehak, and Janssen et al. showed that only hyperhomocysteinemia and anticardiolipin antibodies play a role in the pathogenesis of RVO [6, 17, 23].

## 5. Association of RVO with Thrombophilia and Other Hematologic Risk Factors

Yau et al. recommended that coagulopathy and thrombophilia should also be considered where no obvious RVO etiology is found or if the patient is young or has bilateral RVO, a history of thrombosis, or a family history of thrombosis [7].

Based on RVO in younger patients, risk factors that predispose to coagulation abnormalities have been studied in detail. Published data on thrombophilic abnormalities in RVO patients have accumulated recently but these reports consist of small studies and case reports based on retrospective information on patients retrieved from clinical databases [24–26]. Prospective studies on the association between thrombophilic factors and RVO are rare [9].

The role of thrombophilic risk factors in RVO is still controversial. Various studies have shown conflicting results. High levels of plasma homocysteine and a well-recognized risk factor for arterial and venous thrombotic events, as well as low levels of vitamin B6 and folic acid, have been identified as independent risk factors for RVO [27]. Systemic inflammatory conditions like vasculitis and Behcet disease may increase the risk of RVO [3, 10]. A number of studies have reported that increased blood viscosity, factor V Leiden mutation, hyperhomocysteinemia, and protein C or S deficiency may play a role in the development of CRVO [6, 17, 26, 28, 29]. Glueck et al. in a case control study identified elevated homocysteine and factor V Leiden mutation as risk factors but found no association of anticardiolipin antibodies or lupus anticoagulant with CRVO [28]. Janssen et al. confirmed in a meta-analysis of thrombophilic risk factors that elevated homocysteine and anticardiolipin antibodies seem to be associated with CRVO [30]. Di Capua et al. in a recent large case control study found no association between CRVO and thrombophilic risk factors, including homocysteine levels and anticardiolipin antibodies [31]. Recurrent CRVO has also been reported with elevated homocysteine levels in one study but associations with anticardiolipin antibodies and factor V Leiden mutation were not identified as risk factors in a multivariate study comparing patients with recurrent CRVO to those with one CRVO [31]. Given the conflicting findings, the specific roles of various thrombophilic factors and hypercoagulability in CRVO need further study [9].

## 6. Conclusion

RVO exists in two subtypes: CRVO (central retinal vein occlusion), which is less common and represents occlusion of central retinal vein, and BRVO (branch retinal vein occlusion), which is 4–6 times more common and represents occlusion of some branches of central retinal vein.

RVO is the second most common retinal vascular disease after diabetic retinopathy. RVO is a relatively common and frequently devastating cause of visual loss mainly in older patients. Its prevalence varies according to studies in overall populations from 5.2 to 16 per 1000.

Visual acuity is primarily decreased due to macular edema and retinal ischemia. Although it was first recognized over a century ago, the exact pathogenesis remains unclear, despite a variety of systemic and ophthalmic risk factors that have been identified. A basic risk factor for RVO is advanced age. The risk is further connected with systemic conditions like HTN, arteriosclerosis, DM, HLD, vascular cerebral stroke, blood hyperviscosity, and thrombophilia. The metabolic syndrome (HTN, DM, and HLD) is a strong risk factor for RVO. Individuals with end-organ damage caused by diabetes mellitus and hypertension have strongly increased risk for RVO. Race seems to be also risk factor for RVO. Blacks are more often diagnosed with RVO than non-Hispanic whites. Females are, according to some studies, at lower RVO risk. The role of thrombophilic risk factors in RVO is still controversial. Congenital thrombophilic diseases like factor V Leiden mutation, hyperhomocysteinemia, and anticardiolipin antibodies increase the risk of RVO. Cigarette smoking also increases risk of RVO. Systemic inflammatory conditions like vasculitis and Behcet disease increase the risk. Ophthalmic risk factors for RVO are ocular hypertension and glaucoma, lower ocular perfusion pressure, and congenital and acquired changes in retinal arteries.

## Figures and Tables

**Table 1:** Systemic and ocular risk factors for RVO [5, 7].

Systemic risk factors	Ocular risk factors
Hypertension	Glaucoma

Diabetes mellitus	Decreased ocular perfusion pressure

Hyperlipidemia	External retrobulbar compression-orbital neoplasma and endocrine orbitopathy

Atherosclerotic associated diseases: ischemic heart disease, obesitas (high body mass index), and cigarette smoking	Retinal arteriolar signs-focal arteriolar narrowing and arteriovenous nicking

Systemic vasculitis: systemic lupus erythematosus, sarcoidosis, and syphilis	
Hematologic neoplasia: polycythaemia vera, multiple myeloma, and leukemia	
Hypercoagulation diseases: antifosfolipid syndrome and protein S deficiency	
Drug therapy: oral contraceptives, diuretics, and hypotensive drugs	
